# Implications of allometric model selection for county-level biomass mapping

**DOI:** 10.1186/s13021-017-0086-9

**Published:** 2017-10-18

**Authors:** Laura Duncanson, Wenli Huang, Kristofer Johnson, Anu Swatantran, Ronald E. McRoberts, Ralph Dubayah

**Affiliations:** 1 Biosciences Laboratory, NASA Goddard Space Flight Center, Greenbelt, USA; 2 Department of Geographical Sciences, University of Maryland, College Park, USA; 3 USDA Forest Service, Northern Research Station, Newton Square, PA USA; 4 USDA Forest Service, Northern Research Station, Saint Paul, MN USA

**Keywords:** Forest biomass, Lidar, Allometry, Carbon accounting

## Abstract

**Background:**

Carbon accounting in forests remains a large area of uncertainty in the global carbon cycle. Forest aboveground biomass is therefore an attribute of great interest for the forest management community, but the accuracy of aboveground biomass maps depends on the accuracy of the underlying field estimates used to calibrate models. These field estimates depend on the application of allometric models, which often have unknown and unreported uncertainties outside of the size class or environment in which they were developed.

**Results:**

Here, we test three popular allometric approaches to field biomass estimation, and explore the implications of allometric model selection for county-level biomass mapping in Sonoma County, California. We test three allometric models: Jenkins et al. (For Sci 49(1): 12–35, 2003), Chojnacky et al. (Forestry 87(1): 129–151, 2014) and the US Forest Service’s Component Ratio Method (CRM). We found that Jenkins and Chojnacky models perform comparably, but that at both a field plot level and a total county level there was a ~ 20% difference between these estimates and the CRM estimates. Further, we show that discrepancies are greater in high biomass areas with high canopy covers and relatively moderate heights (25–45 m). The CRM models, although on average ~ 20% lower than Jenkins and Chojnacky, produce higher estimates in the tallest forests samples (> 60 m), while Jenkins generally produces higher estimates of biomass in forests < 50 m tall. Discrepancies do not continually increase with increasing forest height, suggesting that inclusion of height in allometric models is not primarily driving discrepancies. Models developed using all three allometric models underestimate high biomass and overestimate low biomass, as expected with random forest biomass modeling. However, these deviations were generally larger using the Jenkins and Chojnacky allometries, suggesting that the CRM approach may be more appropriate for biomass mapping with lidar.

**Conclusions:**

These results confirm that allometric model selection considerably impacts biomass maps and estimates, and that allometric model errors remain poorly understood. Our findings that allometric model discrepancies are not explained by lidar heights suggests that allometric model form does not drive these discrepancies. A better understanding of the sources of allometric model errors, particularly in high biomass systems, is essential for improved forest biomass mapping.

**Electronic supplementary material:**

The online version of this article (doi:10.1186/s13021-017-0086-9) contains supplementary material, which is available to authorized users.

## Background

Forest aboveground biomass mapping has emerged as a critically important initiative for both constraining the global carbon cycle [18] and facilitating climate mitigation initiatives such as REDD+ [8]. Estimates of aboveground biomass are typically generated through a combination of field sampling and extrapolation using remote sensing data [9]. Lidar remote sensing, in particular, has emerged as a popular technology for mapping aboveground biomass in high biomass systems, as there is no apparent saturation of lidar metrics with high biomass provided that the lidar pulses penetrate to the ground [22]. However, the accuracies of all remote sensing-based biomass maps are inherently dependent on underlying accuracies of the field estimates of biomass that are used to calibrate remote sensing-based models.

Field estimates of biomass are generally estimated through the application of an allometric model relating some measurable attribute of field biomass, e.g. tree stem diameter or height, to aboveground biomass [5, 12]. These allometric models are typically developed through the destructive sampling of a relatively small number of trees, which are directly measured for their biomass. As destructive samples are costly to acquire, the sample sizes used to construct allometric models tend to be relatively small and spatially clustered [7]. As such, the accuracies of these allometric models outside their areas of development remain largely unknown due to a dearth of available sampled validation data [3]. Therefore, determining which allometric model to select for the estimation of field biomass is largely speculative.

In the United States, the two most common sets of allometric models are (a) a set of generalized models developed through a meta-analysis of the literature [12], and (b) the US Forest Service’s Component Ratio Method (CRM) [10, 20]. Jenkins et al. [12] combined thousands of allometric models that performed destructive sampling of trees, and simplified them to just 10 models for general applicability. The Jenkins et al. [12] models have been widely applied in North America, but, as acknowledged by the authors, there are some inherent weaknesses in these models. Notably, the mean number of trees destructively sampled per species in the available Jenkins papers was only 39 [7]. Additionally, the applicability of these models outside the environmental conditions or size class sampled is unknown. In an updated version of these models, Chojnacky et al. [4] included more models from the literature, and used taxonomic groupings and wood specific densities to regroup the original Jenkins divisions, producing 35 generalized models. Although these models are theoretically different, they are still based on the same original models (with some additions) and therefore should produce similar estimates. In contrast, the US Forest Service’s Forest Inventory Analysis program uses an entirely different approach to estimate a tree’s biomass: the CRM, [10, 20]. This method predicts tree merchantable volumes from models based on attributes such as stem diameter, height, and species. Tree volume is then used in conjunction with published wood specific gravity values to estimate the biomass in various components of the tree, namely bole, bark, and branches. Tree biomass is then calculated as the sum of these components.

Past analyses have demonstrated that the Jenkins models produce systematically higher estimates of biomass when compared to CRM, ranging from an 11% difference [13] to a 20% difference [4, 21]. Over the whole coterminous US, the Jenkins models yielded 16% greater biomass than CRM [6]. Although we can only speculate as to which set of allometric models produces a more accurate estimate of field biomass, it is important to characterize the sensitivity of biomass maps to allometric model selection. However, this is not commonly conducted in biomass mapping initiatives. In this study we explicitly test the sensitivity of county-level biomass estimates from lidar to allometric model choice, using the Jenkins et al. [12] models, Chojnacky et al. [4] models, and FIA’s CRM models.

## Methods

As part of NASA’s Carbon Monitoring System (CMS), a pilot project has been funded to map forest aboveground biomass with wall-to-wall airborne lidar over Sonoma County, California. Part of this project has been focused on developing empirical models relating field estimates of forest biomass to lidar metrics, and applying the models to produce county-level biomass maps. The field and lidar data presented in this paper were collected as part of this CMS project.

### Field data

A total of 179 variable radius plots were collected across Sonoma County in 2014. Plot locations were selected through a stratified sampling approach aimed at ensuring a uniform distribution of plots from short (< 5 m), medium (5–25 m) and tall forests (> 25 m), and primarily comprised of conifers, deciduous trees, non-forest, mixed forest, wetlands and an herb and shrub class taken from the Calveg database [15]. Variable radius plots were established, and the diameter at breast height (DBH) and species of all trees in a variable radius plot were recorded, as well as the height of the tallest 1–3 trees in the plot. The plot centroid locations were recorded along with their GPS accuracy. The average centroid GPS location error was 3.45 m. Tree species information is available in Additional file 1: Table S1.

### Field biomass estimation

**Fig. 1:**
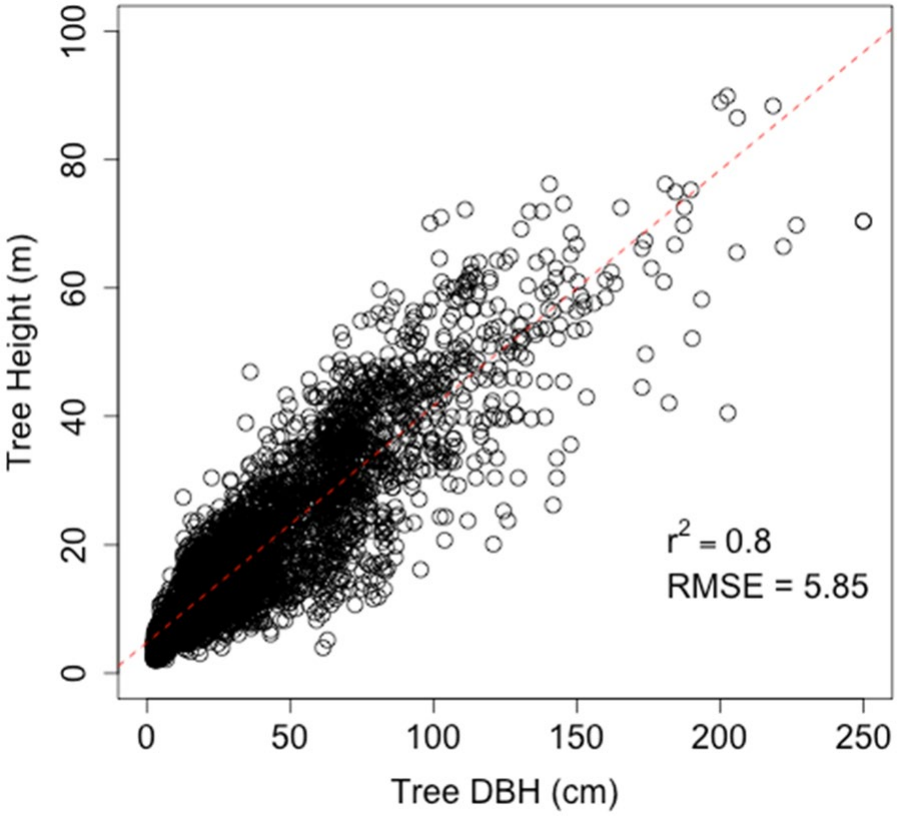
Linear regression predicting tree height as a function of diameter from FIA data in Sonoma County, California

We used three different sets of allometric models to estimate the field biomass in each plot. The first two sets of models, the Jenkins et al. [12] and Chojnacky et al. [4] models, are based on a meta-analysis of published allometric models predicting biomass as a function of DBH and species. The third set follows the CRM, which for the species in Sonoma County requires tree height as a predictor variable [10, 20]. Although we only collected tree heights for a few of the tallest trees in each plot, we were able to estimate biomass with the CRM approach by predicting tree height as a function of DBH. We developed an empirical model predicting tree height as a linear function of DBH from all trees measured in the FIA dataset for Sonoma County for the 2001–2010 period (r^2^ = 0.8, RMSE = 5.85 m, Fig. 1).

We also tested the effects of omitting “cull” by substituting measured tree heights for predicted heights on estimates of county biomass totals, but found no substantial difference. The height prediction model occasionally over-predicted tree heights for large DBHs, when DBHs were outside the range used to calibrate the height model, and we adjusted overestimates by setting the maximum tree height to the maximum height found in each 30 m Lidar pixel. These tree height estimates, as well as tree species and DBH, served as inputs to the CRM estimates of tree biomass.

To estimate plot level biomass, we estimated a biomass density at the plot centroid by summing the biomass estimates for each tree, divided by the ‘plot area’ of that tree. Therefore each tree contributes to the biomass density of the plot centroid as a function of its biomass, and distance from the plot centroid.

### Lidar data

Wall-to-wall lidar data and high-resolution imagery were collected over Sonoma County in the summer of 2014. The lidar data were acquired at 900 m above ground with a field of view of 30°, with a nominal pulse density of 10.66 pulses/m^2^ at 105 kHz. We filtered lidar returns to include only returns from vegetated surfaces. To accomplish this, a tree canopy mask was generated using high-resolution imagery and lidar using an object-based, data-fusion approach [17]. We used LAS tools software to extract vegetated lidar returns within 15 m of field plot centroids, and generate a suite of lidar metrics, including height percentiles, bincentiles (percentage of points between the height cutoff and the maximum height), canopy cover and density, intensities and intensity percentiles, and the quadratic mean lidar height of returns. A radius of 15 m was selected to approximately match the 30 m desired resolution of the county-wide map. We also tested using variable lidar radii to match the variable radius plots used in this study, but we found no statistically significant improvement in model performance (Duncanson et al. in prep).

### Biomass modeling

Random forest regression [1] was applied to model field biomass as a function of lidar metrics (described above) and ancillary metrics, including topography and species composition. We built three different random forest models, one per allometric approach. We used the default random forest values of 500 trees, and 7 input variables (mtry = 7). We filtered outliers from the analysis that had lidar heights greater than 10 m, but a field biomass estimate of zero. We also filtered out four outliers that had small field biomass estimates (< 50 Mg/ha) but high lidar heights (> 30 m), assuming that these plot locations exhibited geolocation errors. We applied these two filters to remove obvious spatial outliers in the dataset where we think the forest canopy mask did not sufficiently remove vegetated returns from tall, non-vegetated surfaces (e.g. buildings). These two filters reduced our sample size from 179 to 166 plots.

### Biomass mapping

We generated three maps of forest biomass density across the county, one for each set of allometric models. We estimated total aboveground biomass for the county and also divided pixels into discrete biomass density categories to assess the differences between allometric approaches in different biomass classes. We estimated the mean for the county by adjusting the mean pixel values to compensate for estimated deviation resulting from systematic model prediction error. We follow the model assisted regression estimator approach outlined by [16], to estimate both mean county biomass and also the standard error of the mean. We used a t test to assess whether county mean biomass estimates were statistically significantly different when using the different allometric models. A t test was selected both for simplicity and because the county-level biomass densities in forested areas of Sonoma County are approximately normal distributions (Fig. 9).

## Results

### Field biomass estimation

**Fig. 2:**
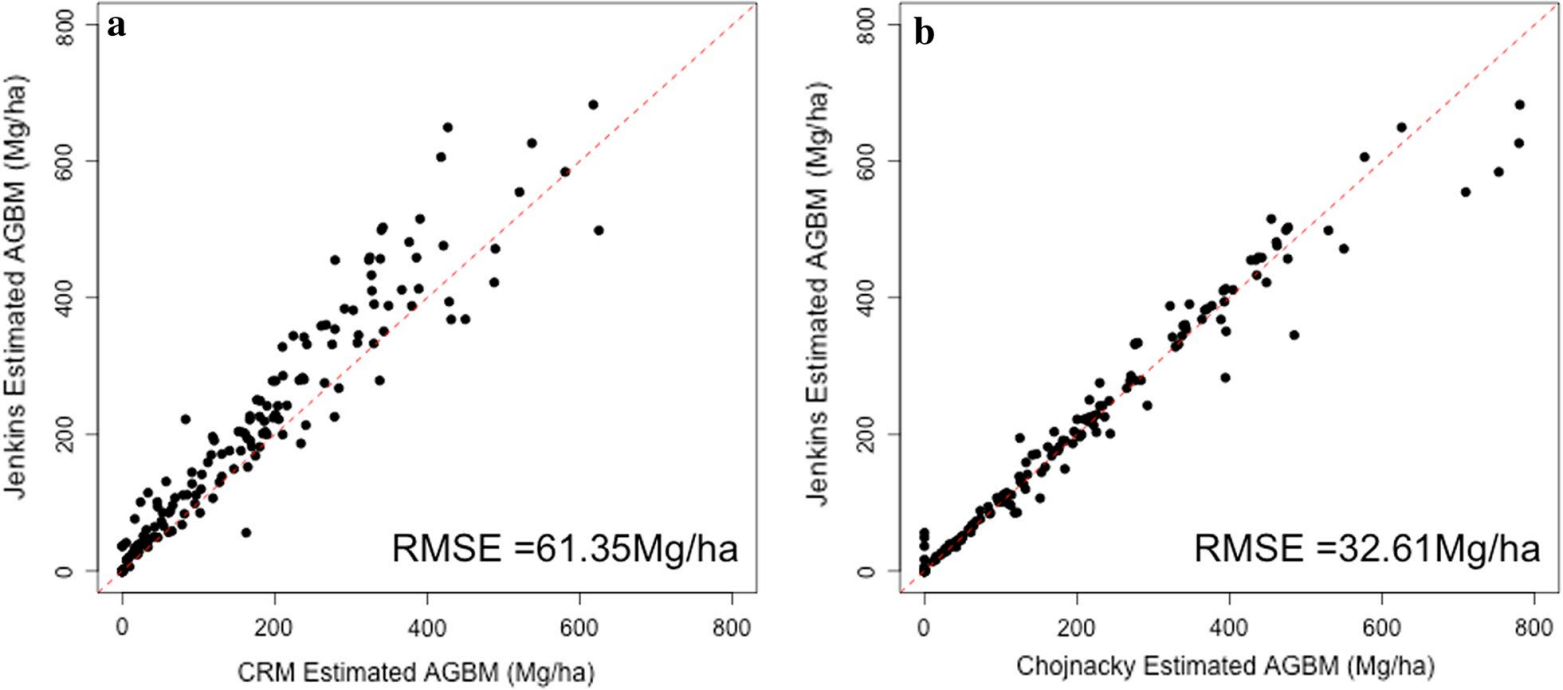
Field plot estimates of aboveground biomass between Jenkins and the FIA’s CRM (**a**) and Jenkins and Chojnacky (**b**) show that FIA’s CRM estimates are generally smaller, with greater differences in high biomass plots, while Jenkins and Chojnacky are comparable although Chojnacky produces greater estimates in large biomass plots. The dotted line represents the 1:1 line

**Fig. 3:**
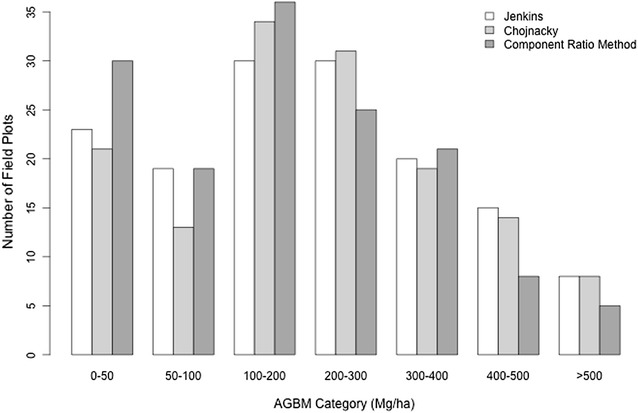
The distribution of field estimates of biomass based on the three allometric approaches. The CRM produced lower estimates of biomass, visualized here as greater numbers of field plots with AGBM < 200 Mg/ha, and fewer large biomass plots, while the Jenkins and Chojnacky approaches produced approximately the same number of estimates per biomass density category. In general, we sampled a larger number of low and medium biomass plots than high biomass plots in this study based on our stratified field sampling approach

At a field plot level, the average biomass density estimates using the Jenkins et al. [12] method, the Chojnacky et al. [4] method, and the FIA’s CRM were 196.1, 195.8, and 164.9 Mg/ha, respectively. Although the average values for Jenkins and Chojnacky are statistically indistinguishable, there is a 19% difference between those estimates and the FIA’s CRM. The difference between Jenkins estimates and CRM estimates is larger in plots with greater biomass values (Fig. 2a). Although the mean Jenkins and Chojnacky estimates match, the plot-level estimates differ appreciably, with Chojnacky estimating greater biomass density values in some high biomass plots (Fig. 2b). The distributions of field estimates from the three approaches show that the shape of the histogram of field plots is captured by all three approaches, although again CRM produces systematically smaller estimates (Fig. 3).

### Biomass density modeling

**Fig. 4:**
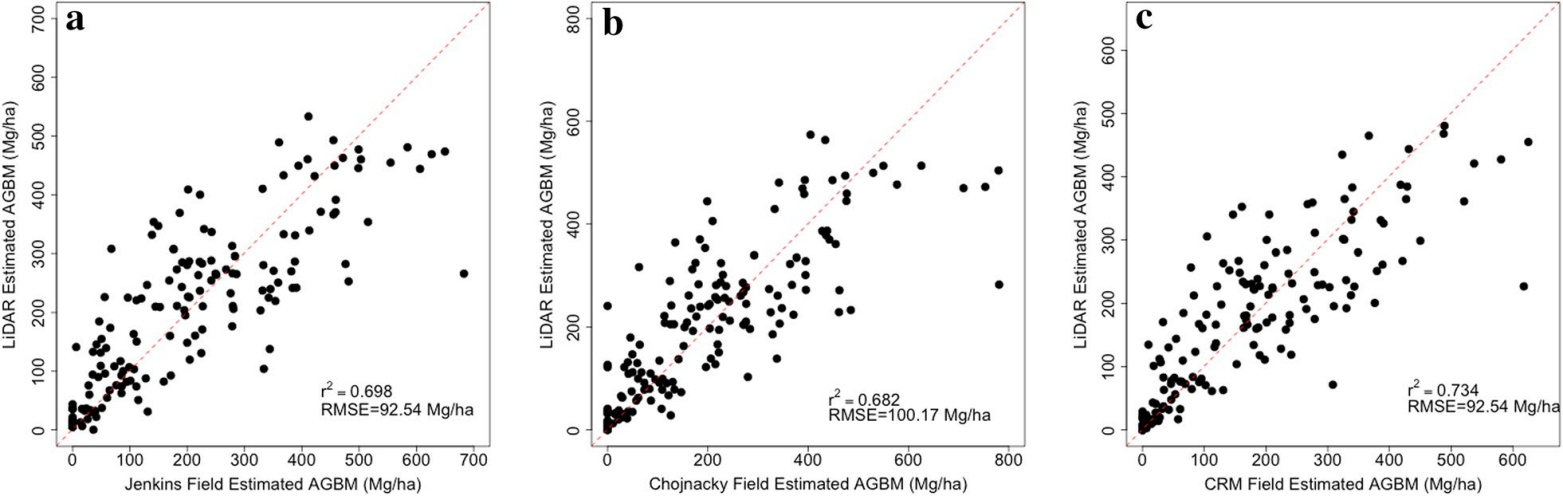
Random forest modeling results predicting field estimates from (**a**) Jenkins, (**b**) Chojnacky and (**c**) FIA’s CRM. All three models performed comparably with slightly better performance by FIA’s CRM method and slightly poorer performance using the Chojnacky estimates

We produced three models of biomass density, one per allometric approach. We found comparable model performance between the three allometric approaches, with the FIA’s CRM producing slightly higher correlations with lidar metrics than either the Jenkins or Chojnacky models (Fig. 4). The RMSE is also slightly lower for the FIA’s CRM, but the %RMSE is similar because of the 19% lower estimates. The %RMSE for Jenkins, Chojnacky, and FIA’s CRM are 46, 50, and 46%, respectively.

Of the 40 predictor variables, the percentile height metrics were the most strongly correlated with above ground biomass. In particular, the lower relative height metrics were more sensitive to biomass (p10, p30, p40) than the higher height metrics. Additionally, the quadratic mean was a good predictor of forest biomass, followed by the higher height percentiles. Indeed, all of the height percentiles were more important predictors than any other variable, apart from the quadratic mean.

### The relationship between Lidar and allometric variability

**Fig. 5:**
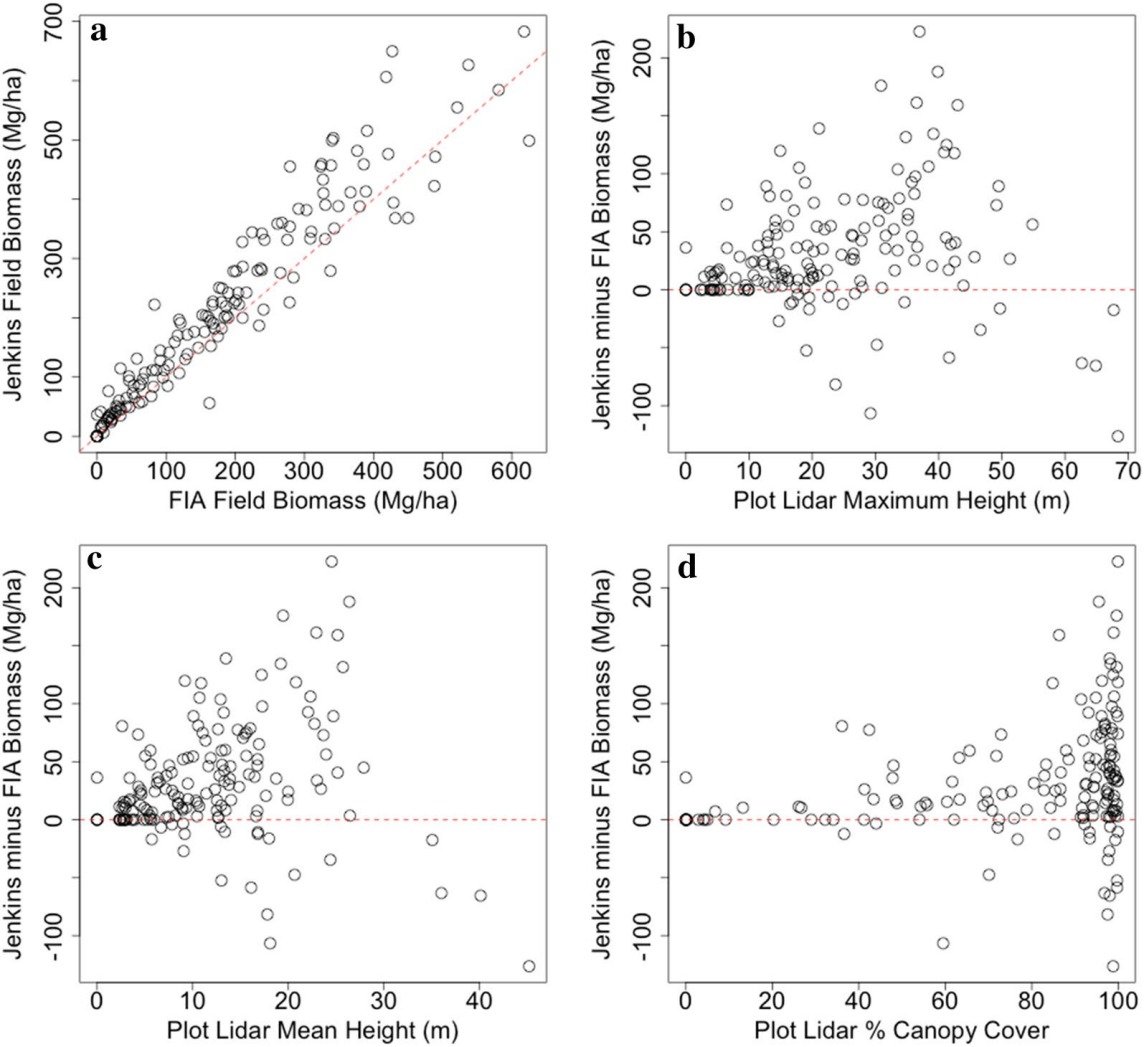
The largest deviations between the FIA and CRM occur in high biomass forests (**a**), with maximum heights between 25 and 45 m (**b**), mean heights between 15 and 30 m (**c**) and canopy cover > 80% (**d**)

**Fig. 6:**
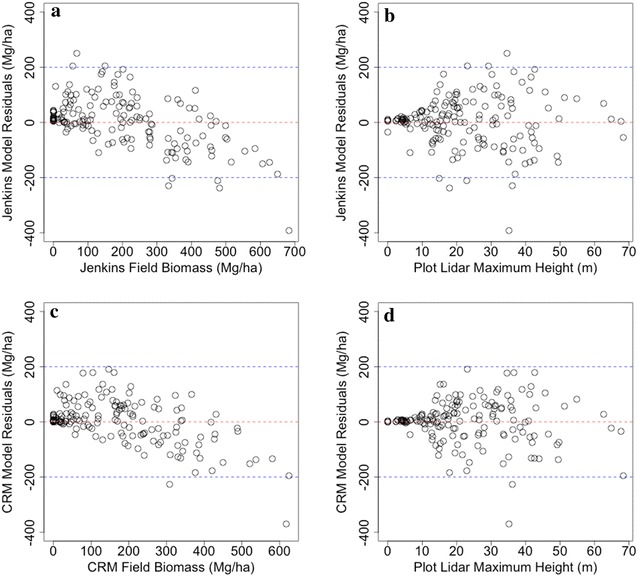
Random forest models relating lidar predicted biomass to field estimates using both Jenkins equations (**a**) and CRM equations (**c**) show that both random forest models yield underestimates of high biomass plots and over estimates of low biomass plots. The residuals from these models (**b**, **d**) show that although residuals increase after ~ 10 m of height there is no marked relationship between height and residuals, suggesting that the models do not increase in error with increasing height. In general, the CRM model has slightly lower total bias

Lidar-derived maximum height, mean height and % canopy cover were compared to discrepancies between the CRM and Jenkins estimates (Fig. 5). Additionally, residuals from the random forests models using the CRM and Jenkins biomass estimates were compared to both biomass and maximum forest height in an attempt to understand the drivers of allometric variability as well as the associated implications for biomass mapping (Fig. 6). There are clear patterns of overestimating low biomass densities and underestimating high biomass densities exhibited in all three random forest models, although these patterns are relatively smaller using the CRM allometric estimates (Table 1). The largest deviations between allometric estimates occurred in height ranges between 25 and 45 m, and the four tallest plots in the study area (height > 60 m) all had higher biomass estimates using the CRM approach. The largest discrepancies are not in the tallest forests, but in the 25–45 m forests with dense (> 80% cover) canopies and high estimated biomass density (Fig. 5). We focused on the discrepancies between CRM and Jenkins as these are the most popular techniques applied, and there was no statistically significant difference between county-level estimates using Jenkins or Chojnacky models.

**Table 1:** Average residuals per height and biomass class using the CRM and Jenkins approaches show that while both approaches overestimate low biomass and underestimate high biomass the Jenkins model has a slightly higher overall deviation as well as markedly higher overestimates in low-moderate biomass plots. This trend is not apparent with respect to height class

	CRM	Jenkins	n
0–10 m	4.26	8.25	35
10–25 m	3.24	6.58	66
25–40 m	6.09	− 5.01	43
40–55 m	− 11.81	− 0.27	18
> 55 m	− 56.19	7.5	4
Total	1.2	2.98	166
0–100 Mg/ha	22.7	33.7	71
100–200 Mg/ha	40.44	52.1	36
200–300 Mg/ha	− 6.89	12.03	25
300–400 Mg/ha	− 59.35	− 75.95	21
> 400 Mg/ha	− 111.55	− 88.37	13
Total	1.2	2.98	166

### Biomass density mapping

**Fig. 7:**
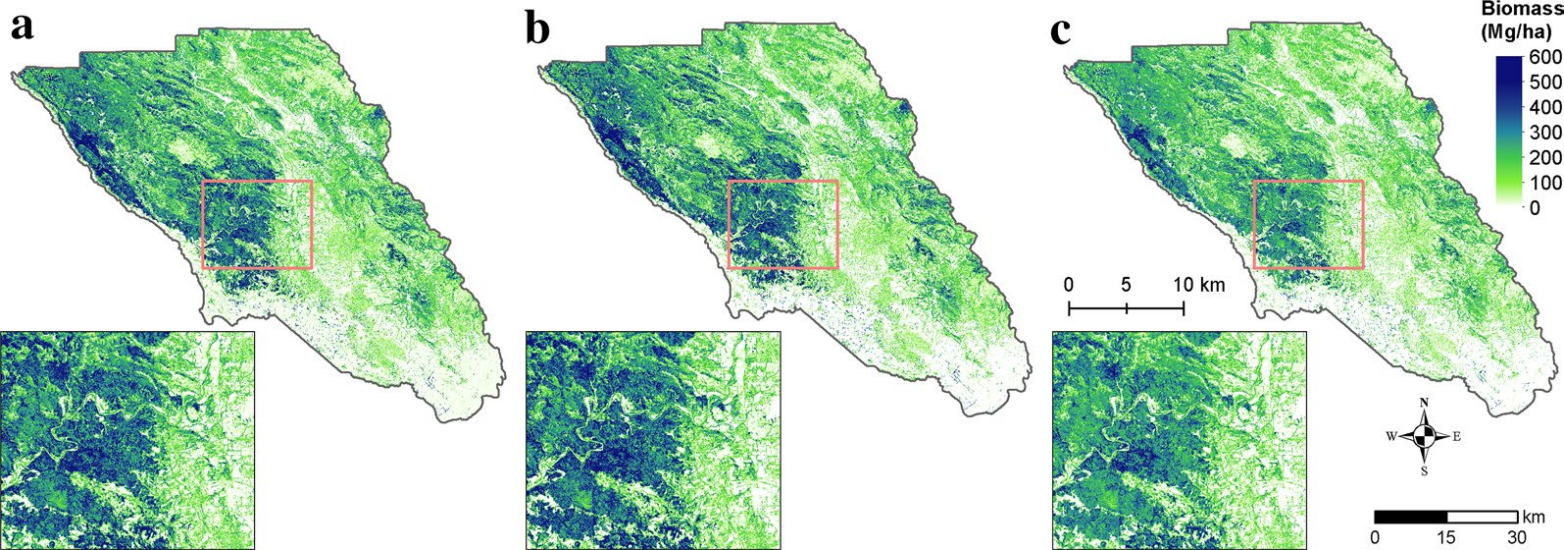
Maps of biomass density predictions for (**a**) the Jenkins approach, (**b**) the Chojnacky approach and (**c**) the CRM approach. The Chojnacky approach produces the greatest estimates in large biomass areas, while the FIA’s CRM approach produces systematically smaller estimates for biomass > 300 Mg/ha

**Fig. 8:**
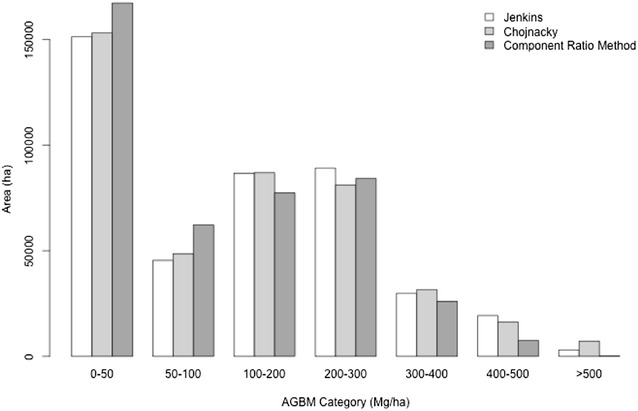
Biomass density distributions by area. Sonoma County is dominated by small biomass areas, with < 4% of the area predicted to have biomass densities > 400 Mg/ha

Each of the three fixed radius biomass models was applied to the full suite of lidar and ancillary metrics for the county at a 30 m resolution. As with the models, the maps show the greatest discrepancies in areas of high biomass (Fig. 7) with Chojnacky predicting the largest high biomass values and the FIA’s CRM predicting the smallest. However, a relatively small fraction of the landscape exists as high biomass (< 4% by area > 400 Mg/ha, Fig. 8), while the majority of Sonoma County’s land area falls either in low biomass (37% in < 50 Mg/ha) or mid-range biomass categories (39% in 100–300 Mg/ha).

**Fig. 9:**
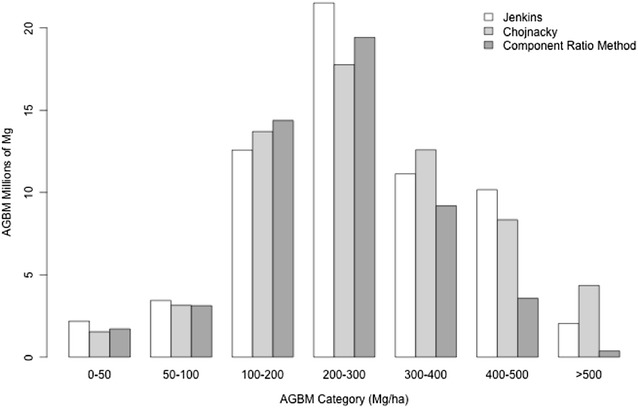
Distributions of total biomass by AGBM category. Predictions in small biomass categories (< 200 Mg/ha) are comparable between the three methods, but smaller predictions > 200 Mg/ha for CRM produce 20% smaller county level estimates. Differences between Jenkins at Chojnacky are apparent in different biomass density categories, but balance over the county to produce the same (< 1% different) county level biomass estimate

The total biomass estimated for Sonoma County for the Jenkins, Chojnacky and CRM approaches was 62.17, 61.72, and 51.62 million Mg, respectively (Table 2). This represents less than 1% difference between the Jenkins and Chojnacky approaches, but a difference of 20.4% between the Jenkins and CRM methods, equating to a difference in 10.55 million tons of carbon. This difference is caused primarily by lower predictions in the high biomass categories (Fig. 9). T tests of the model assisted estimates of the county means, using the model assisted estimators of county variances [16], revealed that the Jenkins and Chojnacky estimates of mean county biomass are not statistically significantly different. Conversely, the CRM estimate of county mean is statistically significantly different (at 95% confidence) from both the Jenkins and Chojnacky estimates.

**Table 2:** Plot and county based statistics describing differences between the three different allometric approaches for plot-level biomass estimation. Total and Mean county estimates are based on map pixels alone, while the model assisted (MA) estimates have been adjusted to compensate for estimated deviation resulting from systematic model prediction (map) error

	Total county (million Mg)	Mean county (Mg/ha)	Mean county (MA, Mg/ha)	SE of mean (MA, Mg/ha)	Mean plot (Mg/ha)	SE residuals (Mg/ha)
Jenkins	62.17	147.8	145.2	7.13	199.2	78.54
Chojnacky	61.72	146.8	143.7	7.73	199.02	85.4
FIA’s CRM	51.62	122.7	121.4	6.09	166.32	68.05

## Discussion

Field estimates of aboveground biomass are often referred to as ‘ground truth’ data in remote sensing studies, but without destructively sampling biomass in the field we do not know how accurate field estimates are. In this study, we demonstrate that allometric model selection yields on average a 19% difference in field plot estimates of aboveground biomass, and a 20% difference in the resulting county-level biomass map for Sonoma County.

Sonoma County is a particularly interesting area to conduct this study, as it hosts forests with some of the highest biomass densities in the United States. We see that the majority of discrepancies between allometric predictions occur in these high biomass areas, which is expected because more trees with smaller stems are destructively sampled for allometric model fitting, and indeed published allometries are often caveated by unknown uncertainties above a certain stem girth [12]. For example, 91 of the trees in our field plots were larger than the largest tree destructively sampled in a respective species class, as reported by Jenkins, thus potentially contributing to greater errors for these 91 trees. Although these trees only represent 8% of those sampled across the county, they represent 18% of the total estimated biomass in our field plots. However, given a lack of other available models, generalized allometric models such as applied in this paper are typically applied regardless of tree size.

The Jenkins and Chojnacky models were expected to perform similarly, as they are largely based on the same datasets of destructively sampled trees. The two studies combined the meta-analysis differently, partitioning allometric models from the literature into different combinations based on generalized species classes [12] or theoretical taxonomic groupings and wood specific gravity [4]. On average, these models produce similar biomass estimates and total county-level predictions, but discrepancies exist on a plot-to-plot basis depending on the species composition of a given plot. Most notably, the Chojnacky models produce greater estimates in high biomass plots, potentially because the models used by Chojnacky are more species-specific than the Jenkins models.

As with previous studies [4, 21], we found that the CRM field plot estimates were ~ 20% less than the Jenkins predictions. In a similar analysis in Maryland, CRM estimates in FIA plots were only ~ 11% less than the Jenkins estimates at the state level [11, 13]. The discrepancy between the differences seen in Maryland and Sonoma County could be due to the prevalence of conifer growth forms and larger trees found in Sonoma County, where we saw that estimates varied more in high biomass than low biomass areas.

There are several explanations for the differences between the Jenkins/Chojnacky and the CRM estimates seen both in this study and consistently observed in regional and national scale studies [5, 6, 14, 19]. First, the sample sizes used to construct the two sets of allometric models are different which could lead to systematic differences. Duncanson et al. [3, 7] demonstrated that allometric parameters are very sensitive to sample size, and that small sample sizes likely lead to an overestimate in biomass for a given DBH. The Jenkins and Chojnacky datasets are, on average, developed with smaller sample sizes than the FIA analysis [7]. These smaller sample sizes yield model deviations because of probable differences in the destructively harvested size distribution, with the inclusion likelihood of large individuals in a sample decreasing with sample size. Similarly, CRM volume models applied to certain species in Sonoma County were developed with destructively harvested trees outside of the region, which may yield errors if trees in Sonoma County are growing in different climate conditions or have different resource limitations than those included in the sample [19].

Finally, others have suggested that the differences between Jenkins and CRM results are due to the inclusion of tree height in the CRM approach, which may better estimate stem volume. However, we do not see strong evidence of this here, as neither maximum nor mean lidar height were highly correlated to differences between CRM and Jenkins field estimates in the tallest forests in our study area. Indeed, the plots with maximum heights between ~ 25 and 45 m had the largest discrepancies, while the tallest trees sampled approached 70 m in height. This may suggest that Jenkins DBH-based estimates may be over estimating biomass in areas of moderate height (~ 25 m) and potentially underestimating biomass in tall forests (> 50 m), while CRM estimates constrain high predictions for a given DBH in relatively short forests and increase estimates for a given DBH in very tall forests. Notably, all of the plots with the highest discrepancies had > 80% canopy cover. As canopy cover is highly correlated to biomass, it is unclear whether the variability in estimates is because of high canopy covers or high biomass densities. Certainly the limited destructive sample for large tree sizes would explain the high biomass density discrepancies, but it is conceivable that destructively harvested trees were also preferentially extracted from open, easily accessible areas with lower canopy covers. This may have caused deviations in allometries in comparison to trees growing in closed canopy systems with relatively taller, smaller crowned individuals.

Our assessment of the drivers of variability between allometric model selection remains speculative. We see that discrepancies are largest in high biomass plots with high canopies covers and moderate heights. Whether these discrepancies are due to inadequate sampling across gradients of biomass, canopy cover or height in either CRM, Jenkins/Chojnacky, or all datasets remains uncertain. Only testing the different allometric approaches against an independent destructively sampled tree dataset can determine the underlying drivers, and such a dataset is currently unavailable for use in this study. However, these results highlight the importance of improving allometric models for biomass mapping. Fortunately, progress is being made in this field, both through the collection of larger destructively harvested tree datasets that can be used to fit improved models (e.g. [5] or through the derivation of new, non-destructively derived allometries based on terrestrial laser scanning (TLS)(e.g. [2]).

## Conclusions

All empirically derived aboveground biomass estimates are fundamentally based on the application of allometric models in the field, and thus have an error that is often unknown and unreported. The allometric models used in this study showed an approximately 20% difference in both mean plot-level and county-level totals of estimated aboveground biomass. This 20% difference is not a 20% error, as we do not have direct field measurements of biomass. Indeed, the error in field biomass estimation is likely to be greater than 20%, particularly in high biomass forests such as exhibited in some areas of Sonoma County. We found the largest discrepancies between allometric field estimates in high biomass plots with heights between 25 and 45 m, with > 80% canopy cover. Lidar heights were not highly correlated to discrepancies amongst popular allometric approaches, suggesting that an incorporation of height into models is unlikely to fully resolve observed discrepancies.

We anticipate many of existing problems related to forest biomass allometry will be addressed by the growing popularity of TLS, which enables field measurement to expand past stem diameters and heights to include full tree volumes. Taken in combination with traditional mensuration, this technology can either directly replace field estimates of biomass through direct estimation of individual tree volumes at the plot level, or improve existing allometric models through the inclusion of much greater numbers of non-destructively sampled individuals.

The findings in this not only underscore the importance of allometry in forest biomass mapping, but highlight that errors in existing allometric models are poorly understood. Further research into the effects of sample size, geographic representativeness, functional form, and the utility of TLS to address these questions is required to properly characterize errors in field estimates of biomass, and propagate these errors through to maps. This is particularly timely considering several upcoming active remote sensing datasets that will be used to map forest biomass at a global scale (e.g. NASA’s GEDI, NISAR, ESA’s BIOMASS). As in this study, the quality of these global maps will depend on the quality of field data used to calibrate the associated empirical biomass models, which will necessarily depend on the accuracy of the underlying allometric models. Thus, forest allometry is not only important at the local–regional scale studies in this paper, but for carbon accounting at a global scale.

## Additional file

**Additional file 1: Table S1.** Tree species sampled in Sonoma County.
