# Supplementary material for: Implications of allometric model selection for county-level biomass mapping
Source: Carbon Balance Manag. 2017 Oct 18;12:18. doi: 10.1186/s13021-017-0086-9 (PMC5647317; doi:10.1186/s13021-017-0086-9)
Supplement: Supplementary file 1 — Additional file 1: Table S1. Tree species sampled in Sonoma County. [file 13021_2017_86_MOESM1_ESM.docx]

Table S1. Tree species sampled in Sonoma County.

| **CLASSIFICATION** | **FAMILY** | **SCIENTIFIC NAME** | **COMMON NAME** |
| --- | --- | --- | --- |
| GYMNOSPERM | Pinaceae | Abies grandis | grand fir |
| DICOT | Sapindaceae | Acer macrophyllum | big leaf maple |
| DICOT | Asteraceae | Achillea millefolium | common yarrow |
| DICOT | Sapindaceae | Acer negundo | box elder |
| DICOT | Sapindaceae | Acer saccharinum | silver maple |
| DICOT | Rosaceae | Adenostoma fasciculatum | chamise |
| FERN | Pteridaceae | Adiantum jordanii | California maidenhair fern |
| DICOT | Sapindaceae | Aesculus californica | California buckeye |
| DICOT | Betulaceae | Alnus rhombifolia | white alder |
| DICOT | Betulaceae | Alnus rubra | red alder |
| DICOT | Boraginaceae | Amsinckia intermedia | fiddleneck |
| DICOT | Myrsinaceae | Anagallis arvensis | scarlet pimpernel |
| DICOT | Apiaceae | Anthriscus caucalis | bur chervil |
| MONOCOT | Poaceae | Anthoxanthum occidentale | sweet grass |
| MONOCOT | Poaceae | Anthoxanthum odoratum | sweet vernal grass |
| DICOT | Ericaceae | Arctostaphylos canescens | hoary manzanita |
| DICOT | Aristolochiaceae | Aristolochia californica | Dutchman's pipe |
| DICOT | Ericaceae | Arctostaphylos columbiana | redwood manzanita |
| DICOT | Asteraceae | Artemisia douglasiana | mugwort |
| MONOCOT | Poaceae | Arundo donax | giant reed |
| DICOT | Araliaceae | Aralia californica | elk clover |
| DICOT | Ericaceae | Arctostaphylos manzanita | whiteleaf manzanita |
| DICOT | Ericaceae | Arbutus menziesii | Pacific madrone |
| FERN | Woodsiaceae | Athyrium filix-femina | common lady fern |
| DICOT | Aristolochiaceae | Asarum caudatum | wild ginger |
| MONOCOT | Poaceae | Avena barbata | slender oat |
| DICOT | Asteraceae | Baccharis pilularis | coyote brush |
| DICOT | Asteraceae | Baccharis salicifolia | mulefat |
| FERN | Blechnaceae | Blechnum spicant | deer fern |
| MONOCOT | Poaceae | Bromus diandrus | ripgut brome |
| MONOCOT | Poaceae | Brachypodium distachyon | false brome |
| MONOCOT | Poaceae | Bromus hordeaceus | soft chess |
| MONOCOT | Poaceae | Bromus laevipes | woodland brome |
| MONOCOT | Poaceae | Bromus madritensis | Madrid brome |
| MONOCOT | Poaceae | Briza maxima | big rattlesnake grass |
| MONOCOT | Poaceae | Briza minor | little rattlesnake grass |
| DICOT | Brassicaceae | Brassica nigra | black mustard |
| MONOCOT | Cyperaceae | Carex brevicaulis | short stem sedge |
| DICOT | Brassicaceae | Cardamine californica | milk maids |
| DICOT | Fagaceae | Chrysolepis chrysophylla | giant chinquapin |
| MONOCOT | Cyperaceae | Carex comosa | bristly sedge |
| DICOT | Aizoaceae | Carpobrotus edulis | ice plant |
| MONOCOT | Cyperaceae | Carex lemmonii | Lemmon's sedge |
| MONOCOT | Cyperaceae | Carex multicaulis | forest sedge |
| MONOCOT | Cyperaceae | Carex barbarae | Santa Barbara sedge |
| MONOCOT | Poaceae | Calamagrostis nutkaensis | Pacific reed grass |
| MONOCOT | Cyperaceae | Carex obnupta | slough sedge |
| DICOT | Asteraceae | Carduus pycnocephalus | Italian thistle |
| DICOT | Rosaceae | Cercocarpus betuloides | mountain mahogany |
| DICOT | Rhamnaceae | Ceanothus cuneatus | buck brush |
| DICOT | Rhamnaceae | Ceanothus divergens | Calistoga ceanothus |
| GYMNOSPERM | Pinaceae | Cedrus libani | Cedar of Lebanon |
| DICOT | Rhamnaceae | Ceanothus jepsonii | musk brush |
| DICOT | Rhamnaceae | Ceanothus parryi | Parry ceanothus |
| DICOT | Asteraceae | Centaurea solstitialis | yellow star thistle |
| DICOT | Rhamnaceae | Ceanothus sonomensis | Sonoma ceanothus |
| MONOCOT | Agavaceae | Chlorogalum pomeridianum | common soap plant |
| DICOT | Asteraceae | Cirsium vulgare | bull thistle |
| DICOT | Ranunculaceae | Clematis ligusticifolia | virgin's bower |
| DICOT | Betulaceae | Corylus cornuta ssp. californica | California hazelnut |
| DICOT | Apiaceae | Conium maculatum | poison hemlock |
| MONOCOT | Poaceae | Cynodon dactylon | Bermuda grass |
| MONOCOT | Poaceae | Cynosurus echinatus | dogtail grass |
| MONOCOT | Poaceae | Rytidosperma penicillatum | hairy oat grass |
| MONOCOT | Apiaceae | Daucus carota | Queen Anne's lace |
| MONOCOT | Themidaceae | Dichelostemma capitatum | bluedicks |
| DICOT | Dipsacaceae | Dipsacum fullonum | Fuller's teasle |
| DICOT | Convolvulaceae | Dichondra donelliana | pony's foot |
| FERN | Dryopteridaceae | Dryopteris arguta | California wood fern |
| DICOT | Chenopodiaceae | Dysphania ambrosioides | Mexican tea |
| MONOCOT | Poaceae | Elymus caput-medusae | medusa head |
| MONOCOT | Poaceae | Elymus glaucus | blue wildrye |
| DICOT | Onagraceae | Epilobium brachyantherum | willowherb |
| FERN | Equisetaceae | Equisetum arvensis | field horsetail |
| DICOT | Boraginaceae | Eriodictyon californicum | California yerba santa |
| DICOT | Myrtaceae | Eucalyptus tereticornis | forest red gum |
| MONOCOT | Poaceae | Festuca arundinacea | tall fescue |
| MONOCOT | Poaceae | Festuca californica | California fescue |
| DICOT | Moraceae | Ficus carica | common fig |
| DICOT | Apiaceae | Foeniculum vulgare | fennel |
| MONOCOT | Liliaceae | Fritillaria affinis | checker lily |
| DICOT | Rhamnaceae | Frangula californica | California coffeeberry |
| DICOT | Oleaceae | Fraxinus latifolia | Oregon ash |
| DICOT | Rosaceae | Fragaria vesca | woodland strawberry |
| DICOT | Rubiaceae | Galium aparine | common bedstraw |
| DICOT | Rubiaceae | Galium californicum | California bedstraw |
| DICOT | Rubiaceae | Galium porrigens | climbing bedstraw |
| DICOT | Ericaceae | Gaultheria shallon | salal |
| DICOT | Geraniaceae | Geranium dissectum | cutleaf geranium |
| DICOT | Fabaceae | Genista monspessulana | French broom |
| DICOT | Rosaceae | Heteromeles arbutifolia | toyon |
| DICOT | Asteraceae | Hemizonia congesta lutescens | yellow hayfield tarweed |
| DICOT | Araliaceae | Hedera helix | English ivy |
| GYMNOSPERM | Cupressaceae | Hesperocyparis macnabiana | MacNab cypress |
| GYMNOSPERM | Cupressaceae | Hesperocyparis macrocarpa | Monterey cypress |
| DICOT | Apiaceae | Heracleum maximum | cow parsnip |
| DICOT | Asteraceae | Heterotheca oregana | Oregon golden aster |
| DICOT | Asteraceae | Helenium puberulum | sneezeweed |
| GYMNOSPERM | Cupressaceae | Hesperocyparis sargentii | Sargent cypress |
| DICOT | Rosaceae | Holodiscus discolor | ocean spray |
| MONOCOT | Poaceae | Holcus lanatus | common velvet grass |
| MONOCOT | Poaceae | Hordeum marinum | Mediterranean barley |
| MONOCOT | Poaceae | Hordeum murinum | mouse barley |
| DICOT | Asteraceae | Hypochaeris glabra | smooth cat's-ear |
| DICOT | Hypericaceae | Hypericum perforatum | Klamath weed |
| DICOT | Asteraceae | Hypochaeris radicata | rough cat's-ear |
| MONOCOT | Iridaceae | Iris douglasiana | Douglas' iris |
| MONOCOT | Iridaceae | Iris macrosiphon | bowltube iris |
| MONOCOT | Juncaceae | Juncus effusus | Pacific rush |
| DICOT | Juglandaceae | Juglans hindsii | California black walnut |
| MONOCOT | Juncaceae | Juncus patens | common rush |
| DICOT | Asteraceae | Lactuca serriola | prickly lettuce |
| DICOT | Lamiaceae | Lavandula stoechas | French lavendar |
| DICOT | Fabaceae | Lathyrus vestitus | common Pacific pea |
| DICOT | Caprifoliaceae | Lonicera hispidula | pink honeysuckle |
| DICOT | Asteraceae | Anisocarpus madioides | woodland madia |
| MONOCOT | Ruscaceae | Maianthemum racemosum | false lily of the valley |
| DICOT | Fabaceae | Melilotus albus | white sweetclover |
| MONOCOT | Poaceae | Melica geyeri | Geyer's onion grass |
| MONOCOT | Poaceae | Melica imperfecta | small flower onion grass |
| DICOT | Lamiaceae | Melissa officinalis | lemon balm |
| DICOT | Lamiaceae | Mentha pulegium | pennyroyal |
| MONOCOT | Poaceae | Melica torreyana | Torrey's melic grass |
| DICOT | Phrymaceae | Mimulus aurantiacus | sticky monkey |
| DICOT | Myricaceae | Morella californica | wax myrtle |
| DICOT | Lamiaceae | Monardella purpurea | serpentine monardella |
| DICOT | Boraginaceae | Nemophila heterophylla | white baby blue eyes |
| DICOT | Fagaceae | Notholithocarpus densiflorus var. densiflorus | tanoak |
| DICOT | Apiaceae | Oenanthe sarmentosa | Pacific parsley |
| DICOT | Oleaceae | Olea europaea | olive |
| DICOT | Apiaceae | Osmorhiza berteroi | sweetcicely |
| DICOT | Oxalidaceae | Oxalis corniculata | yellow sorrel |
| DICOT | Oxalidaceae | Oxalis oregana | redwood sorrel |
| DICOT | Apiaceae | Perideridia kelloggii | Kellogg's yampah |
| FERN | Pteridaceae | Pentagramma triangularis | gold back fern |
| MONOCOT | Poaceae | Phalaris aquatica | harding grass |
| GYMNOSPERM | Pinaceae | Pinus attenuata | knobcone pine |
| GYMNOSPERM | Pinaceae | Pinus contorta ssp. contorta | shore pine |
| DICOT | Fabaceae | Pickeringia montana | chaparral pea |
| GYMNOSPERM | Pinaceae | Pinus muricata | Bishop pine |
| GYMNOSPERM | Pinaceae | Pinus ponderosa var. ponderosa | ponderosa pine |
| GYMNOSPERM | Pinaceae | Pinus radiata | Monterey pine |
| GYMNOSPERM | Pinaceae | Pinus sabiniana | digger pine |
| DICOT | Plantaginaceae | Plantago lanceolata | English plantain |
| DICOT | Polygalaceae | Polygala californica | California milkwort |
| DICOT | Salicaceae | Populus fremontii | Fremont cottonwood |
| FERN | Dryopteridaceae | Polystichum munitum | sword fern |
| DICOT | Rosaceae | Prunus cerasifera | plum |
| MONOCOT | Liliaceae | Prosartes hookeri | drops of gold |
| DICOT | Rosaceae | Prunus spp. | plum |
| GYMNOSPERM | Pinaceae | Pseudotsuga menziesii var. menziesii | Douglas fir |
| FERN | Dennstaedtiaceae | Pteridium aquilinum | hairy brackenfern |
| DICOT | Rosaceae | Pyrus communis | common pear |
| DICOT | Fagaceae | Quercus agrifolia | coast live oak |
| DICOT | Fagaceae | Quercus berberidifolia | scrub oak |
| DICOT | Fagaceae | Quercus chrysolepis | canyon live oak |
| DICOT | Fagaceae | Quercus durata | leather oak |
| DICOT | Fagaceae | Quercus garryana | Oregon white oak |
| DICOT | Fagaceae | Quercus kelloggii | California black oak |
| DICOT | Fagaceae | Quercus lobata | valley oak |
| DICOT | Fagaceae | Quercus wislizeni var. wislizeni | interior live oak |
| DICOT | Ranunculaceae | Ranunculus californicus | California buttercup |
| DICOT | Ericaceae | Rhododendron occidentale | western azalea |
| DICOT | Ericaceae | Rhododendron macrophyllum | western rosebay |
| DICOT | Rosaceae | Rosa canum | dog rose |
| DICOT | Rosaceae | Rosa gymnocarpa | dwarf rose |
| DICOT | Lamiaceae | Rosmarinus officinalis | rosemary |
| DICOT | Polygonaceae | Rumex acetosella | sheep sorrel |
| DICOT | Rosaceae | Rubus armeniacus | Himalaya blackberry |
| DICOT | Polygonaceae | Rumex crispus | curly dock |
| DICOT | Rosaceae | Rubus leucodermis | wild raspberry |
| DICOT | Rosaceae | Rubus spectabilis | salmon berry |
| DICOT | Rosaceae | Rubus ursinus | California blackberry |
| DICOT | Apiaceae | Sanicula crassicaulis | Pacific sanicle |
| DICOT | Salicaceae | Salix exigua | sandbar willow |
| DICOT | Salicaceae | Salix laevigata | red willow |
| DICOT | Salicaceae | Salix lasiolepis | arroyo willow |
| DICOT | Adoxaceae | Sambucus nigra | black elderberry |
| DICOT | Salicaceae | Salix sitchensis | Sitka willow |
| DICOT | Lamiaceae | Salvia sonomensis | Sonoma sage |
| DICOT | Scrophulariaceae | Scrophularia californica | bee plant |
| GYMNOSPERM | Cupressaceae | Sequoia sempervirens | coast redwood |
| DICOT | Rubiaceae | Sherardia arvensis | blue field madder |
| DICOT | Asteraceae | Silybum marianum | milk thistle |
| MONOCOT | Poaceae | Stipa pulchra | purple needlegrass |
| DICOT | Lamiaceae | Stachys rigida | rough hedgenettle |
| DICOT | Caprifoliaceae | Symphoricarpos albus | upright snowberry |
| DICOT | Caprifoliaceae | Symphoricarpos mollis | creeping snowberry |
| DICOT | Apiaceae | Torilis arvensis | hedge parsley |
| GYMNOSPERM | Taxaceae | Torreya californica | California torreya |
| DICOT | Anacardiaceae | Toxicodendron diversilobum | poison oak |
| MONOCOT | Melanthiaceae | Toxicoscordion fremontii | Fremont's star lily |
| DICOT | Fabaceae | Trifolium subterraneum | subterranean clover |
| DICOT | Myrsinaceae | Trientalis latifolia | woodland star |
| DICOT | Lauraceae | Umbellularia californica | California bay |
| DICOT | Urticaceae | Urtica dioica | stinging nettle |
| DICOT | Ericaceae | Vaccinium ovatum | evergreen huckleberry |
| DICOT | Vitaceae | Vitus californica | California wild grape |
| DICOT | Violaceae | Viola ocellata | two-spot violet |
| DICOT | Fabaceae | Vicia sativa ssp. nigra | garden vetch |
| DICOT | Violaceae | Viola sempervirens | redwood violet |
| DICOT | Vitaceae | Vitus vinifera | wine grape |
| DICOT | Hydrangeaceae | Whipplea modesta | modesty |
| FERN | Blechnaceae | Woodwardia fimbriata | chain fern |
| DICOT | Asteraceae | Wyethia angustifolia | narrow-leaf mule ears |
| DICOT | Asteraceae | Xanthium strumarium | rough cocklebur |
| MONOCOT | Melanthiaceae | Xerophyllum tenax | bear grass |
